# Renal health benefits and therapeutic effects of parsley (*Petroselinum crispum*): a review

**DOI:** 10.3389/fmed.2024.1494740

**Published:** 2024-12-12

**Authors:** Sami Alobaidi

**Affiliations:** Department of Internal Medicine, University of Jeddah, Jeddah, Saudi Arabia

**Keywords:** parsley, *Petroselinum crispum*, renal health, nephroprotection, antioxidant, anti-inflammatory, diuretic, clinical trials

## Abstract

Parsley (*Petroselinum crispum*) has been used in traditional medicine for its diverse health benefits, and recent research highlights its potential in promoting renal health due to its bioactive compounds. This review focuses on evaluating the renal health benefits and therapeutic effects of parsley, addressing the growing interest in natural interventions for kidney-related conditions. It aims to consolidate existing evidence while identifying research gaps to support parsley’s integration into renal health management. A systematic review of scientific databases was conducted, identifying relevant studies on parsley’s biochemical properties, including flavonoids, phenolic acids, terpenoids, and essential oils, which contribute to antioxidant, anti-inflammatory, diuretic, and nephroprotective effects. Animal studies demonstrated reductions in oxidative stress, improvements in metabolic biomarkers, and enhanced renal function, while limited human studies revealed modest improvements in urinary composition and renal health markers. However, parsley’s safety profile, particularly at high doses, requires further investigation, including potential herb-drug interactions and safety during pregnancy. This review highlights parsley’s therapeutic potential as a natural agent for renal health and underscores the need for robust clinical trials, long-term safety evaluations, and standardized methodologies to validate its clinical significance.

## Introduction

Kidney health is crucial for maintaining homeostasis by regulating fluid balance, electrolytes, and waste products through urine. Chronic kidney disease (CKD) and other renal conditions pose significant public health concerns, affecting approximately 10% of the global population and often leading to severe outcomes, including end-stage renal disease (ESRD) and cardiovascular complications (1). The prevalence of CKD is rising, largely driven by increases in diabetes, hypertension, and aging populations (2).

Parsley (*Petroselinum crispum*) has a rich history in traditional medicine, primarily known for its diuretic and anti-inflammatory properties. Ancient Greeks and Romans used parsley not only as a culinary herb but also for its medicinal benefits. For instance, it was often prescribed to treat urinary tract infections and kidney stones due to its diuretic properties, which were believed to help flush out toxins and prevent the formation of kidney stones. The Ebers Papyrus, an ancient Egyptian medical document from *circa* 1,550 BC, mentions the use of parsley for its therapeutic properties, particularly for its stimulant and carminative effects. Dioscorides, in his seminal work “De Materia Medica,” noted the use of parsley for its diuretic properties, alongside other medicinal plants (3).

In traditional Chinese medicine, parsley was utilized to treat hypertension and improve digestive health, while Ayurvedic practitioners recommended it for its anti-inflammatory and detoxifying effects (4). Additionally, myristicin, a significant compound in parsley, has been shown to possess various pharmacological activities contributing to its medicinal properties (5).

In recent years, scientific studies have begun to validate these traditional uses, revealing a variety of pharmacological activities attributed to parsley’s bioactive compounds (6). Recent evidence highlights the potential of parsley to improve renal biomarkers and mitigate oxidative stress and inflammation, particularly in experimental and clinical settings. These findings underscore the growing interest in parsley as a complementary intervention for kidney-related conditions. Despite these findings, there remains a need for a comprehensive synthesis of current research specifically focusing on parsley’s effects on kidney health. This review aims to fill this gap by drawing from both human and animal studies. By consolidating findings from these studies, this review seeks to evaluate parsley’s potential as a therapeutic agent for renal protection and provide a comprehensive understanding of its mechanisms of action and safety profile.

## Methods

A comprehensive literature search was conducted to gather relevant studies on the effects of parsley on kidney health and other health benefits. Multiple databases were searched, including PubMed, Scopus, and Web of Science. The search terms used included “parsley,” “*Petroselinum crispum*,” “kidney health,” “renal protection,” “diuretic effects,” “antioxidant properties,” “anti-inflammatory effects,” “clinical studies,” “animal studies,” and combinations of these terms. The search was limited to articles published in English.

Studies were included in the review if they were original research articles, reviews, or clinical trials focused on the effects of parsley on kidney health. Studies investigating the biochemical properties, mechanisms of action, safety, and additional health benefits of parsley were also included. Both human and animal studies were considered, and articles providing quantitative or qualitative data relevant to the topic were selected. Studies were excluded if they were not related to parsley, did not focus on kidney health, were editorials, letters to the editor, opinion pieces without original data, or duplicate publications.

Data extraction involved gathering key information from each study, including study design, sample size, intervention details, outcomes measured, and main findings. The extracted data were synthesized to provide a comprehensive overview of the current state of research on parsley’s effects on kidney health and other health benefits. Similar studies were grouped together, and their findings were summarized to highlight common trends and significant results.

## Biochemical properties of parsley

Parsley is a nutrient-dense herb rich in various bioactive compounds that contribute to its wide range of medicinal properties. The key biochemical constituents of parsley include flavonoids, essential oils, vitamins, and minerals. These compounds are responsible for the herb’s antioxidant, anti-inflammatory, and diuretic effects, among others (4, 6, 7).

### Flavonoids

Parsley contains several flavonoids, such as apigenin, luteolin, and quercetin. These compounds are known for their potent antioxidant and anti-inflammatory properties. Apigenin, for instance, has been shown to induce apoptosis in cancer cells and inhibit oxidative stress (8). Recent studies have identified apigenin-O-pentoside-O-hexoside as a major compound in parsley, significantly contributing to its antioxidant activity (9). Furthermore, apigenin demonstrates antiproliferative activity against A375 human melanoma cells by inducing caspase 3 activity and triggering apoptotic events. Additionally, apigenin has significant radical scavenger capacity, iron chelation potential, and lipoxygenase inhibition activity, highlighting its biochemical potency (10). Luteolin and quercetin also contribute to reducing inflammation and protecting against oxidative damage (11). The bioavailability of apigenin from parsley has been confirmed in human studies, indicating its potential biological effects (12).

### Essential oils

The essential oils of parsley, which include myristicin, apiol, and eugenol, play a significant role in its therapeutic effects. Myristicin has demonstrated various pharmacological activities, such as anti-inflammatory, analgesic, and neuroprotective effects (5). Apiol, traditionally used as a diuretic, also exhibits antimicrobial properties, making parsley effective in treating urinary tract infections (7). Furthermore, parsley essential oil has shown potential in antioxidant and antimicrobial activities (13).

### Furocoumarins

Parsley contains furocoumarins such as psoralen, 8-methoxypsoralen (8-MOP), and 5-methoxypsoralen (5-MOP), which have significant antimicrobial properties. These compounds inhibit pathogens like *Escherichia coli* O157 and *Listeria monocytogenes*, suggesting potential applications in food preservation to prevent microbial spoilage and foodborne illnesses (14).

### Phytoestrogens

Parsley contains significant phytoestrogens, including 6-acetylapiin and petroside, which exhibit estrogen-like activity. These compounds have potential health benefits, particularly in hormone-related conditions, due to their ability to modulate estrogen receptors (15).

### Polyphenol oxidase

Parsley contains the enzyme polyphenol oxidase (PPO), which plays a significant role in its biochemical properties. PPO catalyzes the oxidation of phenolic compounds, contributing to parsley’s antioxidant activity. The kinetic properties of PPO, including its optimal pH, temperature, and substrate specificity, underline its crucial role in neutralizing free radicals and reducing oxidative stress. Inhibition studies have shown that compounds like sodium azide, ascorbic acid, L-cysteine, and glutathione effectively reduce PPO activity, highlighting a mechanism through which parsley mitigates oxidative damage (16).

### Vitamins and minerals

Parsley is an excellent source of vitamins A, C, and K, as well as folate and iron. Vitamin C is a powerful antioxidant that supports immune function and enhances iron absorption. Vitamin K is essential for blood clotting and bone health. Folate is crucial for DNA synthesis and repair, while iron is necessary for oxygen transport in the blood. Parsley also contains other important phytochemicals, such as carotenoids (e.g., beta-carotene) and coumarins. Beta-carotene is a precursor to vitamin A and plays a role in maintaining healthy vision and immune function. Coumarins have anticoagulant properties and may help improve blood circulation (4).

### Chlorophyll

Chlorophyll, the green pigment in parsley, has been shown to possess antioxidant and detoxifying properties. It aids in neutralizing free radicals and protecting cells from oxidative damage. Additionally, chlorophyll has been reported to promote wound healing and support overall detoxification processes (6).

## Mechanisms of action

### Diuretic effects: inhibition of Na+/K + -ATPase

The diuretic effects of parsley are primarily attributed to the inhibition of the Na+/K + -ATPase enzyme in the renal cortex and medulla. This inhibition interferes with potassium secretion and sodium reabsorption, resulting in increased urine output. Studies have demonstrated that parsley extract significantly increases 24 h urine volume in animal models by reducing the activity of Na+/K + -ATPase in both the renal cortex and medulla, leading to reduced apical cellular Na + reabsorption, lower K+ secretion, and increased K+ concentration in the intercellular space (11, 17, 18). Furthermore, compounds such as myristicin and apiol present in parsley enhance this diuretic effect by promoting the excretion of excess salts and water from the body (11, 17, 19–21). The aqueous extract of parsley seeds also increases urinary flow and the excretion of sodium and potassium while lowering arterial pressure in rats, confirming its natriuretic and hypotensive effects (22).

### Antioxidant mechanisms

The antioxidant properties of parsley help reduce oxidative stress, which is crucial in the context of kidney diseases. Flavonoids like apigenin and apiin enhance enzymes such as superoxide dismutase (SOD) and catalase (CAT), neutralizing free radicals and protecting renal tissues (7, 23, 24). Myristicin induces glutathione S-transferase (GST) subunit in the liver, aiding detoxification (25, 26). Polyphenol oxidase (PPO) in parsley also boosts antioxidant capacity by oxidizing phenolic compounds (16). Parsley’s high polyphenol content supports its strong antioxidant activities (27).

Parsley, alone or with *Apium graveolens*, reduces serum uric acid levels, improves renal histopathology, and decreases oxidative stress markers in hyperuricemic rats (28, 29). It protects against renal dysfunction and ischemia/reperfusion-induced acute kidney injury by mitigating oxidative damage and enhancing renal function (30, 31). Parsley extract also improves antioxidant capacity and metabolic biomarkers in high-fat diet-fed and diabetic rats (32, 33). Additionally, parsley reduces oxidative stress in lens tissues and stress-induced gastric oxidative damage by increasing tissue glutathione levels and enhancing SOD and CAT activities (34, 35).

### Anti-inflammatory effects

Inflammation is a critical factor in the progression of kidney diseases. Parsley exhibits significant anti-inflammatory properties, largely attributed to its flavonoid and essential oil content. These compounds reduce levels of inflammatory markers such as tumor necrosis factor-alpha (TNF-*α*) and interleukin-1 beta (IL-1β). Animal studies have demonstrated that parsley extract significantly lowers these inflammatory markers, thereby protecting renal tissues from inflammation-induced damage (31, 36, 37).

Parsley also modulates cytokine production by enhancing anti-inflammatory cytokines such as IL-10 while reducing pro-inflammatory cytokines like IL-1β (38). Additionally, the alcoholic extract of parsley significantly inhibits key inflammatory mediators including nitric oxide (NO) and prostaglandin E2 (PGE2) (39) Recent studies highlight that myristicin and apiol, compounds in parsley, inhibit the production of inflammatory cytokines such as TNF-*α*, IL-1β, and TGF-β1 by blocking their active sites through hydrophobic and hydrogen bond interactions. These findings suggest a robust anti-inflammatory mechanism that could provide additional protection to renal tissues by reducing cytokine-mediated damage (40). Furthermore, myristicin has been shown to inhibit the production of inflammatory mediators like NO, IL-6, IP-10, MCP-1, MCP-3, GM-CSF, MIP-1α, MIP-1β, and LIF in dsRNA-stimulated macrophages, with this inhibition occurring via the calcium pathway, reducing intracellular calcium levels and down-regulating excessive production of these mediators (41). Thus, myristicin effectively mitigates inflammation, offering additional protection to renal tissues.

### Antidiabetic effects

Parsley exhibits significant antidiabetic effects through multiple mechanisms. It reduces blood glucose levels, enhances insulin secretion, and improves glucose metabolism due to flavonoids like apigenin. Parsley extract increases plasma insulin levels and liver pyruvate kinase activity, enhancing insulin function. Its potent antioxidant properties reduce oxidative stress markers and enhance antioxidant enzyme activities, protecting pancreatic *β*-cells from damage. Additionally, parsley’s anti-inflammatory properties help mitigate inflammation, further supporting insulin function and glucose metabolism. These combined effects underscore parsley’s potential as a complementary treatment for diabetes (33, 42–44).

### Antimicrobial and antifungal activities

Parsley’s antimicrobial properties are attributed to its essential oils, which contain bioactive compounds like monoterpenes, apiol, and myristicin. These compounds inhibit the growth of various bacterial and fungal strains. Recent research highlights that parsley essential oil effectively prevents fungal growth, particularly Aspergillus flavus, in food products like cheese, demonstrating its potential for use in food preservation (45). Additionally, studies show significant antibacterial activity against both Gram-positive and Gram-negative bacteria, and antifungal activity against *Candida albican* (9, 13).

Furocoumarins identified in parsley, such as psoralen, 8-methoxypsoralen (8-MOP), and 5-methoxypsoralen (5-MOP), also exhibit antimicrobial properties, inhibiting pathogens like *Escherichia coli* O157 and *Listeria monocytogenes*. These findings suggest potential applications of parsley in food preservation, although further investigation into the toxicity of these compounds is needed (14). The dual antioxidant and antibacterial properties of parsley, as reported by Wong and Kitts (46), further support its potential use as a natural preservative and antimicrobial agent.

### Antithrombotic effects

Parsley extract exhibits significant antithrombotic properties attributed to its rich content of flavonoids and phenolic compounds, such as apigenin, cosmosiin, and apiin. These bioactive compounds inhibit platelet aggregation and adhesion to collagen, which are critical steps in thrombus formation. Studies have demonstrated that parsley extract and its purified flavonoids significantly reduce platelet aggregation induced by ADP and thrombin in both *in vitro* and *ex vivo* models (47–49). Furthermore, oral administration of parsley extract in rat models increased carotid artery occlusion time and reduced venous thrombus formation, indicating its potential effectiveness in preventing both arterial and venous thrombosis (50, 51).

### Hypolipidemic effects

Parsley’s hypolipidemic effects are attributed to its ability to inhibit lipid peroxidation and enhance the activity of antioxidant enzymes such as superoxide dismutase (SOD) and catalase (CAT). These effects are mediated by bioactive compounds like glucosinolates, betalains, and phenolic compounds, which protect against oxidative stress and improve lipid profiles. Studies have shown that methanol extract of parsley seeds significantly reduces serum levels of total cholesterol, triglycerides, low-density lipoprotein (LDL), and very low-density lipoprotein (VLDL), while increasing high-density lipoprotein (HDL) in hypercholesterolemic rats. These findings suggest that parsley can effectively manage hyperlipidemia and reduce the risk of cardiovascular diseases (52, 53).

### Estrogenic activity

Recent studies have demonstrated significant estrogenic effects of parsley extracts, evidenced by increased uterine protein levels and serum estradiol levels. The hydro-ethanolic extract and polyphenolic fraction of parsley exhibited significant estrogenic activity, contributing to its potential benefits in addressing female infertility (54). Additionally, parsley root extract (PCE) interacts with estrogen receptors, indicating its potential role in managing hormone-dependent conditions (15, 55).

### Enhancement of lysosomal biogenesis and cellular repair

A study by Helmy et al. (56) demonstrated that combined extracts of gum Arabic, parsley, and corn silk increased lysosomal biogenesis in mice renal cells. This enhancement promotes cellular repair mechanisms crucial for maintaining renal health and function. The study highlighted that parsley, as part of the combined extracts, contributed to the reparative mechanisms in renal cells, suggesting its potential role in protecting against renal cell damage and promoting recovery from acute kidney injury (56).

### Anxiolytic and antidepressant effects

Parsley polyphenols exhibit significant anxiolytic and antidepressant effects through multiple mechanisms. The phenolic compounds, including ferulic acid, cinnamic acid, quercetin, and hydroxytyrosol, possess potent antioxidant properties that neutralize free radicals and reduce oxidative stress, protecting neural tissues. These polyphenols also modulate neurotransmitters such as serotonin, dopamine, and norepinephrine, which play crucial roles in mood regulation. Additionally, their anti-inflammatory effects reduce pro-inflammatory cytokines, alleviating inflammation in the brain. Furthermore, parsley polyphenols enhance neuroplasticity by promoting the growth and survival of neurons, thereby improving mood and cognitive function (57).

### Gastroprotective mechanisms

Parsley exhibits significant gastroprotective effects through multiple mechanisms. It enhances gastric mucus secretion, providing a protective barrier against gastric acid and other harmful agents, thus maintaining the integrity of the gastric epithelium and preventing ulcer formation. Parsley also has direct protective effects on gastric tissues, reducing histopathological damage scores. Additionally, its rich antioxidant content, including flavonoids, carotenoids, and ascorbic acid, supports the cellular defense system by neutralizing reactive oxygen species (ROS) and mitigating oxidative damage. These combined mechanisms work synergistically to protect the gastric mucosa from damage and prevent the development of gastric ulcers (35, 58).

### Anticancer properties

Parsley exhibits significant anticancer properties through multiple mechanisms. It induces apoptosis and inhibits cell proliferation in human melanoma and breast cancer cells. The essential compounds, including apigenin and other polyphenols, contribute to its antiproliferative activity. In particular, the dichloromethane extract of parsley has demonstrated substantial antioxidant activity, reducing DNA damage and inhibiting cancer cell migration (8, 10). Additionally, parsley root extract significantly inhibits DNA synthesis, metabolic activity, and cell proliferation in both benign and malignant mammary cells, showing promise as a potential anticancer agent (55).

### Spasmolytic effects

Parsley extract exhibits significant spasmolytic effects on rat ileum tissue, particularly under varying calcium chloride concentrations. The muscle relaxant effect of parsley was dose-dependent and more pronounced at higher calcium chloride concentrations, suggesting its potential therapeutic use in gastrointestinal disorders involving smooth muscle spasms (59).

## Clinical studies on renal protection

### Clinical human studies on renal protection

Parsley has been the subject of several clinical studies investigating its potential benefits for renal health. These studies have explored various aspects, including its impact on urinary composition, antioxidant biomarkers, and renal health markers in different populations. A 2024 study by Essa et al. demonstrated that parsley seed-supplemented bread improved renal biomarkers in obese women, suggesting its potential for broader dietary applications in renal health management (38). The findings from these studies, as presented in Table 1, suggest that parsley may play a role in enhancing renal function and reducing the risk of kidney-related issues, although results are mixed.

**Table 1:** Key findings from clinical human studies on parsley and renal health.

Study	Participants	Intervention	Key findings
Alyami and Rabah (2011) (67)	20 healthy volunteers	Drinking parsley leaf tea	No significant difference in various urinary parameters compared to control group
Nielsen et al. (1999) (11)	14 individuals	Daily intake of parsley	Increased urinary apigenin excretion and enhanced antioxidant enzyme activity (SOD, GPx). Supports potential antioxidant defense and kidney health benefits of parsley.
Essa et al. (2024) (38)	60 Obese women	Parsley seed-supplemented bread	Improved serum osteopontin levels and other renal health markers. Indicates potential improvement in renal function in obese individuals.

While some studies highlight the potential benefits of parsley as a natural therapeutic agent for renal protection, including enhancements in antioxidant defenses and improvements in certain renal health markers, others report no significant changes in urinary parameters. These mixed results indicate the need for further research to conclusively determine parsley’s efficacy in renal health. Limitations such as small sample sizes and specific population groups may affect the generalizability of findings. These aspects are further explored in the limitations section of this manuscript to provide a comprehensive understanding of the current state of research on parsley and renal health.

### Animal studies on renal protection

Animal studies have played a crucial role in understanding the effects of parsley on renal health. These studies provide insights into the mechanisms by which parsley exerts its protective effects and offer preliminary evidence for its potential therapeutic applications. A summary of the key findings from these studies is presented in Table 2.

**Table 2:** Summary of key findings from animal studies on parsley and renal protection.

Study	Animal model	Key findings
Rezazad and Farokhi (2014) (30)	Female Wistar rats	Reduced renal dysfunction induced by prostadin, improved biochemical and histopathological parameters.
Vranješ et al. (2016) (17)	NMRI albino mice	Significant diuretic and antioxidant properties beneficial for kidney function and oxidative stress.
Al-Yousofy et al. (2017) (19)	Rats	Reduced renal oxidative stress and improved renal histology, showing potential antiurolithiatic effects.
Rahmat et al. (2018) (29)	Sprague–Dawley rats	Reduced serum uric acid levels and improved liver and kidney structures in hyperuricemic conditions.
Ajebli and Eddouks (2019) (36)	Wistar rats	Showed antihypertensive activity by inhibiting vascular calcium channels in hypertensive and normotensive rats.
Takrooni et al. (2019) (37)	Male Wistar rats	Ameliorated CsA-induced nephrotoxicity, improving biochemical, oxidative stress, and histopathological markers.
Roshankhah et al. (2019) (31)	Male Wistar rats	Protected against ischemia/reperfusion-induced renal injury by reducing oxidative stress and inflammation.
Helmy et al. (2020) (56)	Male albino mice	Combined extracts of gum Arabic, parsley, and corn silk promoted lysosomal biogenesis and cellular repair in amikacin-damaged renal cells
Soliman et al. (2020) (28)	Male Swiss mice	Ameliorated hyperuricemia-induced renal dysfunction and inflammation, improving biochemical and histopathological parameters.
Essa et al. (2024) (68)	Male Sprague–Dawley rats	Protected against ethylene glycol-induced renal stones, improved renal function and oxidative stress markers.
Mohamed et al. (2024) (40)	Rats	Demonstrated significant reduction in oxidative stress and inflammatory markers in the liver and kidneys, with histopathological evidence supporting hepato-renal protection.

Parsley’s antioxidant properties, diuretic effects, and potential to ameliorate nephrotoxicity have been extensively studied in animal models. Key studies have demonstrated that parsley extract can protect against renal dysfunction, enhance antioxidant defenses, and improve renal function markers. For instance, Rezazad and Farokhi (30) showed that parsley extract protects against prostadin-induced renal dysfunction in female rats, while Helmy et al. (56) highlighted the role of parsley in promoting lysosomal biogenesis and cellular repair in renal cells. Adding to this evidence, a 2024 study by Mohamed et al. highlighted parsley’s role in reducing oxidative stress and inflammation in rat models, further supporting its therapeutic potential in preventing hepato-renal damage (40).

These studies collectively highlight the potential of parsley as a natural therapeutic agent for renal protection. The benefits observed include improvements in antioxidant defenses, renal function, fluid balance, and protection against drug-induced nephrotoxicity. However, these studies also have limitations, such as small sample sizes, short intervention periods, and species differences, which may affect the generalizability of the findings to humans. Detailed limitations and considerations will be discussed in the limitations section of this manuscript. to provide a comprehensive understanding of the current state of research on parsley and renal health.

Future research should explore the long-term effects of parsley supplementation, investigate its mechanisms of action in greater detail, and conduct studies in more diverse animal models to validate these findings.

## Safety and adverse effects

Understanding the safety profile of parsley, especially at high doses, is crucial. Many studies have evaluated the medicinal properties of parsley and found it to be generally safe when consumed in moderate amounts. However, potential toxic effects, herb-drug interactions, and safety during pregnancy are important considerations.

### General safety profile

Overall, parsley is considered safe for consumption in moderate amounts. Reviews have concluded that parsley has a high safety margin when consumed in dietary amounts with minimal adverse effects at typical consumption levels. For example, studies examining the effects of parsley on metabolic biomarkers in high-fat diet-fed rats reported no significant adverse effects on liver or kidney functions (32). Similarly, parsley extract demonstrated protective effects on renal function in prostadin-induced renal dysfunction with no significant adverse effects at administered doses (30). Long-term administration of hydro-ethanolic extract and polyphenolic fraction of parsley showed no hepatotoxicity or nephrotoxicity in animal studies, indicating a favorable safety profile (54). Additionally, parsley extract improved reproductive outcomes and reduced complications in diabetic pregnancies, demonstrating its safety and protective role during pregnancy (60). Acute toxicity tests indicated a high safety margin for parsley extract with no significant adverse effects at therapeutic doses (58). The bioavailability study of apigenin from parsley also supports its safe consumption, as a small portion of apigenin from parsley reaches the human circulation without adverse effects (12). Furthermore, parsley extract showed no significant cytotoxic effects on the metabolic activity or membrane integrity of SH-SY5Y neuroblastoma cells, reinforcing its non-cytotoxic nature (61).

### Potential toxic effects at high doses

While parsley is generally safe, high doses can lead to toxic effects. High doses of parsley extract (1,000 mg/kg body weight) led to significant increases in liver enzymes, indicating liver damage, and elevated blood urea nitrogen (BUN) and creatinine levels, suggesting kidney toxicity. Histopathological examination revealed inflammatory and necrotic changes in liver and kidney tissue (62). High doses of parsley also caused significant changes in kidney histology and elevated oxidative stress markers in mice, indicating potential toxicity (56). Additionally, Ozsoy-Sacan et al. (63) found that the ethanolic extract of parsley leaves caused significant hematological and biochemical changes in rats, further highlighting the risks associated with high-dose consumption. Bergapten (5-methoxypsoralen), a phototoxic compound in parsley, is more concentrated in the leaves than stems. While ingestion of bergapten from parsley (0.5–0.8 mg per meal) is unlikely to cause photosensitivity, handling parsley followed by sun exposure may cause mild photocontact dermatitis or photopigmentation (64).

### Potential interactions and special considerations

Herb-drug interactions and safety during pregnancy are important considerations for parsley consumption. A case report documented elevated sirolimus levels in a renal transplant patient after consuming large amounts of parsley juice. The study suggested that parsley contains bioactive compounds that can inhibit cytochrome P450 3A4 (CYP3A4) and P-glycoprotein (P-gp) enzymes involved in the metabolism and transport of sirolimus (65). Additionally, a review on herbal medicinal products during pregnancy highlights potential risks such as uterine contractions and possible teratogenic effects associated with parsley consumption during pregnancy (66). These findings underscore the need for caution when consuming parsley alongside critical medications or during pregnancy.

## Comprehensive health benefits of parsley

Parsley has been extensively studied for its potential health benefits beyond renal protection. The bioactive compounds in parsley contribute to its antioxidant, anti-inflammatory, and protective effects across various organ systems. Studies have demonstrated its cardiovascular, antidiabetic, lipid metabolism, hepatoprotective, immunomodulatory, neuroprotective, anti-fatigue, antiosteoporotic, antimicrobial, antianemic, reproductive health, antithrombotic, and antihemolytic properties. A detailed summary of these studies is provided in Table 3.

**Table 3:** Summary of comprehensive health benefits of parsley.

Health benefit	Description
Cardiovascular and antithrombotic benefits	Reduces cardiovascular tissue damage, improves biochemical markers, inhibits platelet aggregation, reduces platelet adhesion, and prolongs bleeding time (47–51, 69, 70).
Antidiabetic effects	Reduces blood glucose, improves pancreatic function, and enhances insulin function. Significant reduction in blood glucose levels and improvement in metabolic biomarkers in diabetic rats (33, 42, 43, 71).
Effects on lipid metabolism	Improves lipid profiles in hypercholesterolemic conditions, significantly reducing serum levels of total cholesterol, triglycerides, LDL, and VLDL while increasing HDL (52, 53)
Hepatoprotective properties	Reduces liver damage, showing antioxidant, anti-inflammatory, hypoglycemic, and hypolipidemic properties (63, 69, 72–74)
Gastroprotective effects	Reduces stress-induced gastric oxidative damage and prevents ethanol-induced gastric ulcers by supporting the cellular antioxidant defense system and reducing histopathological damage (35, 58).
Immunomodulatory effects	Suppresses splenocyte proliferation and nitric oxide production, indicating potential modulation of immune responses (75).
Neuroprotective benefits	Protects against cadmium-and morphine-induced neurotoxicity (76–78)
Anti-fatigue effects	Improves fatigue indicators and regulates gut microbiota, suggesting benefits in reducing fatigue (79)
Antiosteoporotic properties	Improves bone density and oxidative stress markers in glucocorticoid-induced osteoporosis, exhibits protective action against ovariectomy-induced osteoporosis by restoring bone strength and reducing bone resorption and oxidative stress biomarkers (80, 81)
Antimicrobial and antifungal activities	Exhibits significant antibacterial and antifungal activities against various bacterial and fungal strains, including both Gram-positive and Gram-negative bacteria (9, 13, 14, 45, 46).
Antianemic effects	Ameliorates phenylhydrazine-induced anemia in rats by improving blood parameters, preventing hemolysis, and increasing serum iron and ferritin levels (82).
Reproductive health	Exhibits significant protective effects on reproductive health, including protection against acrylamide-induced reproductive toxicity, enhancement of sperm parameters, estrogenic effects benefiting female infertility, protection against testicular damage from toxins, and improvement of reproductive outcomes in diabetic pregnancies (54, 60, 83, 84).
Anticancer properties	Induces apoptosis, inhibits cell proliferation in human melanoma and breast cancer cells, reduces DNA damage, inhibits cancer cell migration, and shows antiproliferative and cytotoxic effects on estrogen receptor-positive mammary cells (15, 24, 55).
Dermatological benefits	Reduces melasma severity with fewer side effects compared to hydroquinone cream (85).
Anxiolytic and antidepressant effects	Reduces depressive and anxiolytic behaviors (57).
Antihemolytic effects	Significant antihemolytic activity, reducing oxidative damage to red blood cells (27).

## Limitations and future research

### Gaps in current research: need for more extensive human clinical trials

While numerous studies highlight the health benefits of parsley, most existing research is based on animal models or *in vitro* studies. More extensive human clinical trials are needed to validate these findings in human populations. These trials should explore the long-term effects of parsley consumption on kidney health and other physiological systems to ensure a comprehensive understanding of its therapeutic potential and safety profile (11, 38).

### Safety evaluations and isolation of active compounds

Parsley contains various bioactive compounds, including flavonoids, terpenoids, and essential oils. Future research should focus on isolating and identifying these active compounds to understand their specific roles and mechanisms of action. This could enhance the development of more targeted therapeutic applications and improve the efficacy of parsley-based treatments. Additionally, comprehensive safety evaluations are needed to determine the long-term safety profile of these compounds (6, 62).

### Exploration of parsley’s effects in combination with other treatments

The potential synergistic effects of parsley when used with other treatments have not been extensively explored. Research should investigate how parsley interacts with conventional medications and other natural remedies to enhance therapeutic outcomes. This includes studying its effects in combination with diuretics, antihypertensives, and anti-inflammatory agents to optimize treatment strategies for kidney and other health conditions (4, 65).

### Estimating dosages in parsley research

One significant challenge is the lack of standardization in parsley preparations and dosages across studies. Variability in preparation methods—such as fresh leaves, teas, decoctions, or pharmacologically quantified supplements—complicates direct comparisons. Observational studies provide valuable insights into real-world intake, but pharmacologically controlled studies are essential to determine therapeutic thresholds and optimize dosing strategies. For instance, Nielsen et al. (11) demonstrated inconsistent effects on ROS in a short-term study, while Alyami and Rabah (67) highlighted the challenge of unquantified active compounds in parsley tea. These examples illustrate the broader need for standardized study protocols, accurate quantification of active substances, and clearly defined clinical endpoints to improve consistency and reliability across studies. Addressing these challenges through standardized protocols and rigorous dosage studies will be critical in ensuring parsley’s therapeutic potential is both reliable and replicable in clinical applications.

### Recommendations for future research

1. **Conduct Human Clinical Trials**: Future studies should focus on conducting extensive human clinical trials to validate findings from animal and *in vitro* studies. These trials should aim to assess parsley’s effects on key clinical endpoints, such as renal biomarkers and oxidative stress markers, in larger, more diverse populations. Standardization of dosages and preparation methods (e.g., fresh leaves, teas, decoctions, or supplements) should also be prioritized to ensure consistency and comparability of findings.
2. **Identify Active Compounds and Evaluate Safety**: Efforts should be made to isolate and identify the specific bioactive compounds in parsley to understand their roles and mechanisms of action, along with comprehensive safety evaluations to determine their long-term safety profiles.
3. **Explore Combination Treatments**: Studies should examine the synergistic effects of parsley when used in combination with other treatments, such as diuretics, antihypertensives, and anti-inflammatory agents.

## Conclusion

In conclusion, parsley demonstrates a wide array of health benefits, making it a valuable addition to both diet and medicinal practices. Research highlights parsley’s potent antioxidant, anti-inflammatory, nephroprotective, and antimicrobial properties, which contribute to its therapeutic potential in various health conditions. The bioactive compounds in parsley, including flavonoids like apigenin and kaempferol, essential oils such as apiol and myristicin, and other phytochemicals, play a crucial role in mediating these beneficial effects.

Parsley has shown promising results in reducing oxidative stress, improving metabolic biomarkers, enhancing renal function, and inhibiting the growth of various bacterial and fungal strains. These findings suggest its potential in preventing and managing kidney-related disorders, infections, and other chronic conditions associated with oxidative damage and inflammation. Recent human and animal studies further support parsley’s role in improving renal health markers and urinary composition. However, large-scale, well-designed trials are still needed to validate these effects in diverse populations.

It is essential to consider the limitations of current research, including the need for more extensive human clinical trials, long-term safety evaluations, and the isolation and characterization of active compounds. Research on pharmacokinetics and standardization of preparations is particularly necessary to optimize therapeutic efficacy. Future studies should also explore the synergistic effects of parsley in combination with other treatments to maximize its therapeutic potential.

Overall, parsley’s multifaceted health benefits underscore its importance as a natural therapeutic agent. Incorporating parsley into dietary interventions and herbal medicine regimens could provide a valuable strategy for enhancing overall health and preventing disease.
